# Sinoatrial Beat to Beat Variability Assessed by Contraction Strength in Addition to the Interbeat Interval

**DOI:** 10.3389/fphys.2018.00546

**Published:** 2018-05-18

**Authors:** Helmut Ahammer, Susanne Scheruebel, Robert Arnold, Michael Mayrhofer-Reinhartshuber, Petra Lang, Ádám Dolgos, Brigitte Pelzmann, Klaus Zorn-Pauly

**Affiliations:** ^1^Institute of Biophysics, Medical University of Graz, Graz, Austria; ^2^KML Vision OG, Graz, Austria; ^3^Institute for eHealth, Graz University of Applied Sciences, Graz, Austria

**Keywords:** heart rate variability, beat to beat variability, video motion analysis, sinoatrial node, acetylcholine, sample entropy, Higuchi dimension

## Abstract

Beat to beat variability of cardiac tissue or isolated cells is frequently investigated by determining time intervals from electrode measurements in order to compute scale dependent or scale independent parameters. In this study, we utilize high-speed video camera recordings to investigate the variability of intervals as well as mechanical contraction strengths and relative contraction strengths with nonlinear analyses. Additionally, the video setup allowed us simultaneous electrode registrations of extracellular potentials. Sinoatrial node tissue under control and acetylcholine treated conditions was used to perform variability analyses by computing sample entropies and Higuchi dimensions. Beat to beat interval variabilities measured by the two recording techniques correlated very well, and therefore, validated the video analyses for this purpose. Acetylcholine treatment induced a reduction of beating rate and contraction strength, but the impact on interval variability was negligible. Nevertheless, the variability analyses of contraction strengths revealed significant differences in sample entropies and Higuchi dimensions between control and acetylcholine treated tissue. Therefore, the proposed high-speed video camera technique might represent a non-invasive tool that allows long-lasting recordings for detecting variations in beating behavior over a large range of scales.

## Introduction

Heart rate variability (HRV) refers to variations in the time intervals between two consecutive heart beats and serves as a diagnostic and prognostic tool for cardiac as well as non-cardiac diseases, e.g., heart failure, aging, Parkinson's disease, diabetes, and sepsis (Goldberger et al., 2002; Devos et al., 2003; Kudat et al., 2016; de Castilho et al., 2017; Elstad et al., 2018; Sessa et al., 2018). These variations are mainly attributed to dynamic changes of neuroendocrine inputs on ion channel activity in the sinoatrial node SAN, but a certain degree of beat to beat variability is inherently present at the level of the isolated heart, within the isolated SAN and also at the level of single sinoatrial pacemaker cells (Lombardi and Stein, 2011; Papaioannou et al., 2013; Yaniv et al., 2014a; Zaniboni et al., 2014).

There is a large number of quantitative algorithms to investigate these interval variations in autorhythmic cardiac tissue, cell clusters, or single cells, including spectral, linear, and nonlinear methods. Power-law behavior of beat to beat intervals *BBIs* analyzed by the power spectral method has been shown for neonatal rat cardiomyocytes in cultured tissue layers measured by microelectrode arrays (Ponard et al., 2007). Long-range correlations were also detected in extracellular electrograms of human embryonic stem cell-derived cardiomyocyte clusters by using again spectral methods (Mandel et al., 2012). Furthermore, fractal-like behavior has been reported for rabbit sinoatrial node tissue and for a small percentage of single sinoatrial node cells by using power law and detrended fluctuation analysis (Yaniv et al., 2014a) and in small clusters of chick embryonic cardiomyocytes (Ahammer et al., 2013). So far, investigations have focused on variabilities in the time domain of both, electrical and contraction signals. The underlying processes are tightly linked via the excitation-contraction-coupling (Eisner et al., 2017) and hence, the time structure of the electrical process substantially shapes not only the frequency of contraction but also its magnitude. Thus, it is reasonable to assume that also the variability of the contraction strength shows long-term correlations.

Therefore, in this study we propose the investigation of contraction strengths and their variabilities additionally to interval variabilities in SAN tissue. In detail, we evaluated beat to beat interval variabilities and beat to beat contraction strength *CS* variabilities of murine atrial preparations that contained the SAN region by means of high-speed camera video recordings. Each image of a video represented a time stamp and contractions of the tissue were recorded as changes in average gray values. Simultaneous measurements of extracellular potentials using a cardiac-near-field electrode validated beat to beat intervals of video recordings. Measurements of the spontaneous activity of tissue samples were performed before and after the administration of acetylcholine ACh, the predominant transmitter of the parasympathetic nervous system. Its effects on atrial tissue are already well investigated and include a decrease in beating rate and force of contraction (Kitazawa et al., 2009).

Our main objectives were to determine the suitability of video recordings to register *BBIs* and *CSs* and to analyze changes of nonlinear measures in the variabilities of these two parameters due to ACh treatment. Sample entropy and Higuchi dimension are popular estimators capturing intrinsic nonlinear patterns in time series of measured signals (Higuchi, 1988; Richman and Moorman, 2000). We hypothesized that ACh significantly affects sample entropies and Higuchi dimensions of *BBI* and *CS* variabilities. To distinguish actual values from white noise, surrogate data series were constructed and analyzed.

In summary, this high-speed camera video recording-technique provides a promising tool to thoroughly investigate beat to beat behavior regarding absolute values of beating rate and contraction strength as well as their variabilities in autorhythmic tissue.

## Methods

### Tissue preparation

Hearts from 22 C57/BL6 wildtype mice (aged 12–20 weeks) of both sexes in equal number were used for this study. The preparation of atria including the intact SAN region was carried out as previously described (Torrente et al., 2015). Briefly, mice were heparinized and anesthetized with ketamine (100 mg/kg) and xylazine (10 mg/kg) and the hearts were quickly removed. The atria including the intact SAN region were dissected from the ventricles and fixed with needles on a silicone ground of an experimental chamber. For this study, an extracted SAN tissue of one mouse represented a single experiment. Therefore, the number of experiments corresponds to the number of mice.

The experimental procedure and number of used animals were approved by the ethics committee of the Federal Ministry of Science, Research and Economy of the Republic of Austria (BMWFW-66.010/0101-WF/V/3b/2016). The experiments were conducted according to the Directive of the European Parliament and of the Council of September 22, 2010 (2010/63/EU).

### Video acquisition

The experimental chamber containing the intact SAN tissue was mounted on the stage of an upright microscope (Olympus, BX51W1, 4x objective, light source TH4-200) and the tissue was superfused with oxygenated standard external solution (containing in mM: NaCl 137, KCl 5.4, CaCl_2_ 1.8, MgCl_2_ 1.1, NaHCO_3_ 2.2, NaH_2_PO_4_ 0.4, HEPES/Na^+^ 10, D(+)-glucose 5.6, pH 7.4 adjusted with NaOH) which was kept at a constant temperature of 23°C. Recordings were started 20 min after the onset of superfusion in order to allow the tissue to establish and maintain a stable beating rhythm. Close to the primary pacemaking site of the tissue, a small image region of interest ROI showing distinct contractions was selected for recording. After recording of the first video (Con), acetylcholine (ACh, 3 μmol/L) was added to the perfusion solution and after 5 min superfusion time the second video was recorded. A number of nine tissue samples yielded 18 videos.

Tissue samples under investigation showed a beat to beat interval of about 500 ms (~2 Hz). In order to measure such intervals, it is necessary to sample the temporal course of the beating with enough data samples per second. The Nyquist-Shannon sampling theorem with a sample rate that is the twofold of the highest frequency in the signal is not applicable, because the content of harmonic frequencies are not of interest. More important is, that the sample rate determines a minimal jitter between consecutive time stamps. This jitter must be small, because it influences the nonlinear analysis including variability measures. For an accuracy of e.g., 1%, a number of 100 data samples is needed between two succeeding beats. Particularly, this would yield a sample rate of 200 Hz. Please note that video acquisitions using standard frame rates of 30 Hz would yield an accuracy of only ~6.67% for a beating rate of ~2 Hz. We decided to set the accuracy to 0.2% and consequently a sample rate of 1 kHz was used.

**Figure 1:**
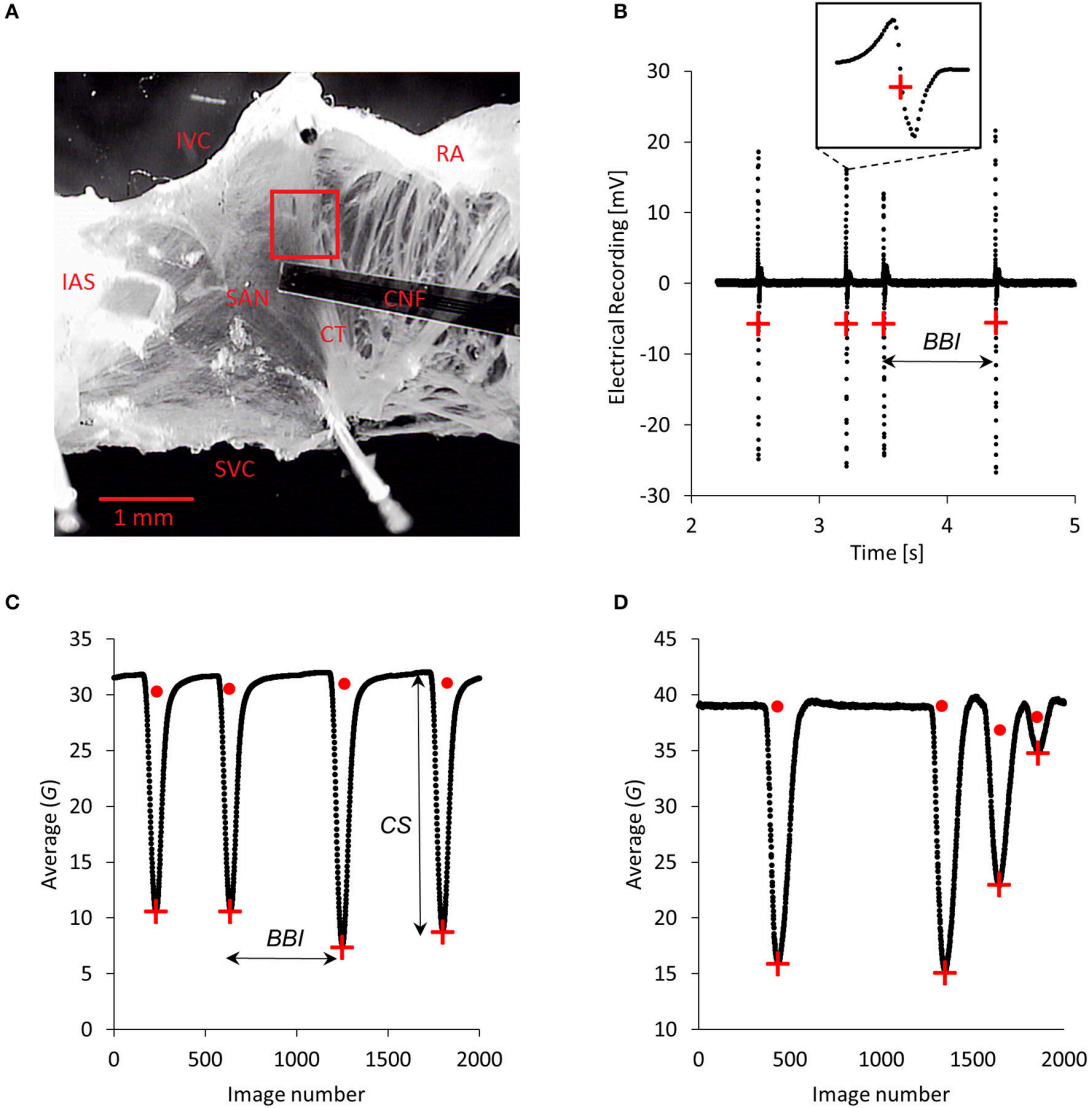
Microscopic sample image and sample recordings. **(A)** Sample image of a tissue preparation including the intact atria and the region of the sinoatrial node SAN. The left atrium cannot be seen in the image. RA, right atrium; CT, crista terminalis; IAS, interatrial septum; IVC, inferior vena cava; SVC, superior vena cava; CNF, cardiac-near-field electrode. The region of interest ROI (red rectangle) with a size of 160 × 160 pixels was set to a region yielding high changes of average gray values. This was usually the case when regions of a thick tissue layer (e.g., trabeculae) moved into the ROI during contractions. **(B)** Sample electrical recording lasting about 4 s (down-sampled to 5 kHz for the graphical representation). Four subsequent beats are depicted and the red plus symbols indicate data points for computing beat to beat intervals *BBIs*. A single beat is zoomed out. **(C)** Sample average gray values $\bar{G}$ according to Equation (1) from about 2,000 (out of 300,000) images. Red plus symbols mark data points that defined the *BBIs* and in addition with the baseline points (red dots) the contraction strengths *CSs*. Interval as well as contraction strength variations are clearly visible for these four contractions. **(D)** Finding correct baseline points is crucial for the determination of the *CSs*. This signal sample shows some beats (red plus symbols) and baseline points (red dots). The first two baseline points were found very well, in contrast to the last two baseline points. Accordingly, *CSs* for these two points cannot be accurately computed.

Video recordings of beating tissue samples were taken by using a high-speed camera system (MotionBlitz, GMCLTR1.3CL-SSL, Mikrotron, Germany) and a video camera (EoSens CL, MC1362, Mikrotron). This system implemented a hardware recording unit and therefore, avoided erroneous jitter effects of software trigger events such as e.g., USB camera solutions do usually show. The resolution of the camera was set to 1,280 × 1,024 pixels. A ROI with a pixel size of 160 × 160 was selected to get maximal gray level changes during beating of the tissue. Figure 1A shows a sample image.

The size of the ROI was empirically optimized by inspections of the temporal signals gained. Larger ROIs yielded too large and inconvenient video files and smaller ROIs yielded too low signal to noise ratios. Regions with thick tissue layers (trabeculae, crista terminalis) moving into the ROI turned out to yield the highest signal to noise ratios. With a sample rate of 1,000 fps and a recording duration of 5 min, a number of 300,000 single uncompressed images (each of them with 160 × 160 = 25,600 pixels) where taken and stored on hard disk in an avi container format. One avi file needed 21 GB of memory. The Mikrotron system saved the individual images in RGB format although the used camera was a gray value camera. Gray value cameras usually give higher signal to noise ratios than color cameras which is important for high-speed acquisitions with very small exposure times. Thus, images were converted in a first step to 8 bit, lowering the memory demand to 7, 6 GB per video. The whole measuring setup was tested against electrical and optical inferences coming from ambient light sources such as the laboratory light or the microscope light source itself. Fourier analyses of videos capturing a static scene revealed no residual frequency components of power supply frequencies or other additional noise components.

### Electrical recordings

For comparison of video and electrical measurements we carried out 13 separate control experiments in order to simultaneously record video as well as extracellular electrical signals. After positioning of the microscope's objective and choosing a ROI, a cardiac-near-field CNF electrode (Hofer et al., 2006) was placed close to the ROI (Figure 1A). This ensured that the electrical and optical measurement sites corresponded in the spatial domain. For evaluation of beat to beat intervals, only one of the four CNF channels was used. Electrical signals were amplified (gain 100), anti aliasing lowpass filtered (4th order Bessel, cutoff frequency 20 kHz) and recorded with custom software (LabVIEW, National Instruments, Austin, Texas) at a sampling rate of 100 kHz (NI USB-6210, National Instruments, Austin, Texas). Signals were digitally filtered (Butterworth lowpass, 4th order, cutoff frequency 1.5 kHz and Butterworth highpass, 4th order, cutoff frequency 1.5 Hz). A sample electrical recording can be seen in Figure 1B. Subsequent time stamps of individual beats were computed by setting a threshold well above the noise level to the decreasing slopes of the signal (denoted by red plus signs in Figure 1B). For signals with lower signal to noise ratio the same threshold criterion was used in the first temporal derivative of the signal where the steep downslope during electrical activation was more pronounced.

### Time signal generation from videos

Time signals of the beating tissues were reconstructed by computing the average gray value ${\bar{G}}_{i}$ of each image *i* of a video which can be seen in Figure 1C.

(1) $$\begin{array}{l} {\bar{G}}_{i}=\frac{1}{N_{P}}\sum_{p=1}^{N_{P}}g_{i,p}, \end{array}$$

with *g*_*i, p*_ the gray value in the range [0, 255] of pixel *p* in the image *i*, *i* = 1, 2, 3, …, *N*_*I*_, *N*_*I*_ the number images in a video (*N*_*I*_ = 300, 000), and *p* = 1, 2, 3, …, *N*_*P*_, *N*_*P*_ the number of pixels in an image (*N*_*P*_ = 25, 600).

**Figure 2:**
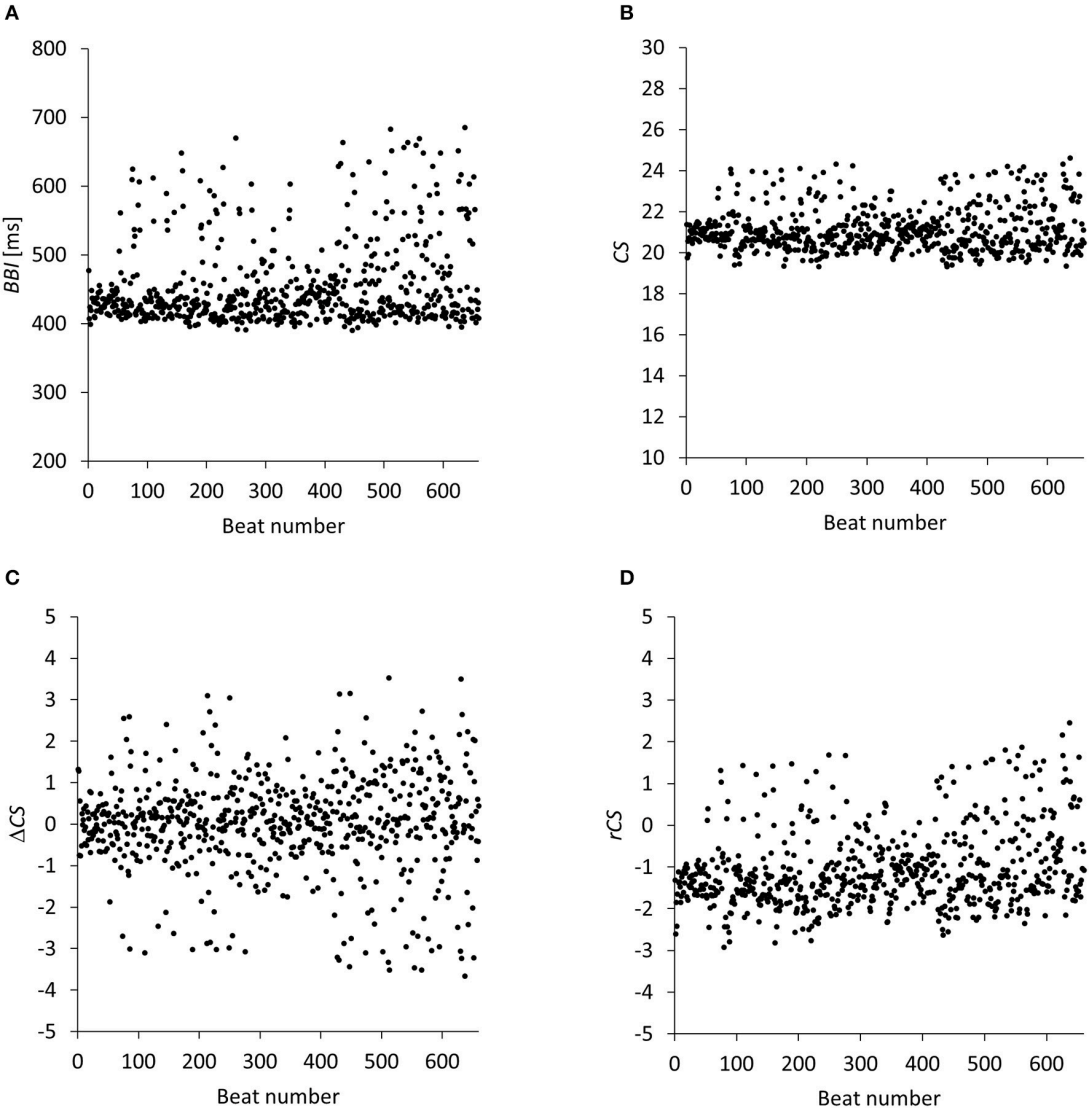
Beat to beat intervals *BBIs* and contraction strengths *CSs*, Δ*CSs*, *rCSs* computed from optical (video) recordings. **(A)** *BBIs* computed according to Equation (2). This tissue sample yielded actually 660 data points (number of beats *N*_*B*_ = 661). **(B)** Contraction strengths *CSs* computed according to Equation (3) for the same tissue sample with 660 data points. **(C)** Differences of contraction strengths Δ*CS* computed as the differences of the average gray values $\bar{G}s\;$ of the beating signal according to Equation (4). **(D)** Relative contraction strengths *rCSs* computed by integration of the Δ*CS* values according to Equation (5).

This yielded a temporal time signal comprising 300,000 data points with a data compression of (25,600 to 1). The algorithm for finding the time stamps of contractions was designed around finding subsequent local minima (denoted by red plus signs in Figure 1C). The original signal was slightly smoothed by applying a moving average filter with 25 data points to improve the shape of the minima and to ensure that minima are right between the adjacent declining and rising slopes. A threshold was set between the doubled noise value of the baseline and the smallest minimum in the video. Further on, only values smaller than that threshold were investigated. Then, a minimum was computed by simply looking for the smallest value between two threshold points. Very rarely a minimum consisted of two neighboring points with exactly the same value in which case the second value was taken as the minimum. Finally, a beat to beat interval *BBI* was defined as the temporal interval between two succeeding minima (beats).

(2) $$\begin{array}{l} BBI_{b}=t_{b}-t_{b-1}, \end{array}$$

with *t*_*b*_, the absolute time of the beat *b* (minimum) in a video, *b* = 2, 3, …, *N*_*B*_, and *N*_*B*_ the number of beats in a video. This algorithm (named PointFinder) was implemented in the software IQM (Kainz et al., 2015) and is available from the authors or from the IQM project page (https://sourceforge.net/projects/iqm/). A sample result of *BBIs* is shown in Figure 2A.

The number of data points in such a graph was equal to the number of beats *N*_*B*_ − 1 during the recording interval of 5 min and therefore, was not constant from video to video. The sample graph in Figure 2A consists of actually 660 data points because the specific tissue sample contracted 661 times in 5 min. Roughly, this reflects another data compression of 450 to 1. Overall the method has a compression rate of about 11.6 million to 1.

On further inspection of Figure 1C, it is obvious that not only interbeat intervals can be computed from these local minima. Additionally, the height of a minimum measured from the baseline, which is the horizontal line containing the points of relaxed tissue only, can be computed. Such a height reflects the mechanical contraction strength *CS* of the tissue. Stronger contraction of cells in the tissue yielded a higher light absorption and hence darker images in the video and consequently lower minima in the average graphs such as in Figure 1C. We created correlation plots of subsequent height changes vs. subsequent interbeat intervals (actual plots are described in section Correlation of Contraction Strengths and Beat to Beat Intervals). A correlation between these two variables was given in most cases, which is in accordance with previously published data (Torres and Janssen, 2011). Nevertheless, correlations were not perfect and some experiments showed only weak or negative correlations. Consequently, we decided to additionally evaluate variations of contraction strengths.

First, in order to obtain the *CS*, it was necessary to determine the baseline, although it drifted during the time course of 5 min. To avoid drifting and offset errors we computed a separate baseline value for each minimum. The actual baseline value was computed by the median of all points in between the actual minimum and the preceding minimum and thus we obtained moving baseline values (for every minimum a separate baseline value, some sample baseline values are depicted graphically in Figure 1C with red points). This ensured that baseline drifts, unavoidable during a recording time of 5 min, did not contribute. Consequently, *CS* was computed by

(3) $$\begin{array}{l} CS_{b}=|{\bar{G}}_{b}-baseline_{b}|, \end{array}$$

with *CS*_*b*_ the contraction strength of the beat *b*, ${\bar{G}}_{b}\;$ the average gray value of the image detected as the “beat image,” *baseline*_*b*_ the corresponding baseline value, *b* = 1, 2, 3, …, *N*_*B*_, and *N*_*B*_ the number of beats in a video. A sample signal can be seen in Figure 2B.

Although the signals seemed to be reliable, it turned out that this moving median algorithm produced some variation errors due to residual noise components of the baseline. Additionally, but only occasionally, for one mouse treated with acetylcholine (#5 in **Figure 6**), subsequent contractions showed some consecutively fast repeating bursts instead of full contractions. For such short burst intervals, the median algorithm yielded erroneous baseline values leading to too small *CS* values. A graphical representation of such errors is shown in Figure 1D. An alternative algorithm for finding the baseline may be feasible, but we present a convenient way that does not need baseline detection at all.

In this approach we eliminated the baseline (offset), since we actually were interested in variabilities of these temporal signals and not in absolute values. Discrete differentiation of the time signal (average gray values according to Equation (1) and exemplarily depicted in Figure 1C) revealed differences of contraction strengths Δ*CSs* and eliminated the hassle of finding baseline points.

(4) $$\begin{array}{l} \Delta {CS}_{b}={\bar{G}}_{b}-{\bar{G}}_{b-1}, \end{array}$$

with b = 2,3,…, *N*_*B*_.

Subsequent discrete integration generated back the changing content of the contraction strength signal but without the baseline and was termed relative contraction strength *rCS*.

(5) $$\begin{array}{l} rCS_{b}=\Delta {CS}_{b}+rCS_{b-1}, \end{array}$$

with *b* = 2, 3, …, *N*_*B*_ and *rCS*_1_ = 0.

Integration usually gives the anti-derivative plus an unknown constant, which was in our case the baseline (offset). Discrete differentiation followed by integration was actually carried out with software IQM (Kainz et al., 2015) using the mathematics feature for one dimensional signals.

Sample graphs of Δ*CS* and *rCS* can be seen in Figures 2C,D. *BBI, CS* and *rCS* data series for each experiment (Con and ACh treated) are provided as “Data Sheet 1” csv file in the supplement.

### Sample entropy and higuchi dimension

Sample entropy *SampEn* and Higuchi dimension *D*_*H*_ are two well-known and successfully applied nonlinear descriptors for time signal variations (Higuchi, 1988; Richman and Moorman, 2000). Approximate entropy *ApEn* is also widely used, but is not suitable for this particular study because the number of beats changed from video to video. *SampEn* is proportional to the conditional probability that a sequence which is similar for *m* points remains similar for *m*+1 points. A tolerance distance *r* is defined so that repetitions must not be exact. Usually, *r* is defined as a multiple of the standard deviation *SD* of the signal and therefore, *SampEn* is a scale invariant measure (Richman and Moorman, 2000). Self matches are not included.

The discrete time signals {*x*(1), *x*(2), …, *x*(*N*_*B*_)} (*x* stands for values from *CS* or *rCS* signals) with length *N*_*B*_ were taken and (*N*_*B*_ – *m* + 1) sequences were created:

(6) $$\begin{array}{l} X_{m}\left(i\right)=\;\left[x\left(i\right),\;x\left(i+1\right),\ldots ,\;x\left(i+m-1\right)\right]. \end{array}$$

The parameter *m* was set to two (*m* = *2*). Distances in between these data series were computed using the maximum metric:

(7) $$\begin{array}{l} d\left[X_{m}\left(i\right),X_{m}\left(j\right)\right]=\underset{k=1,2,..,m}{\max}\left\{\left|X_{m}\left(i+k-1\right)-X_{m}\left(j+k-1\right)\right|\right\} \end{array}$$

The normalized sums of distances smaller than the tolerance distance *r* = 0.15*SD* were computed for each *i, j* with 1 ≤ *i, j* ≤ *N*_*B*_ − *m* + 1 and *i* ≠ *j*:

(8) $$\begin{array}{l} C_{i}^{m}\left(r\right)=\frac{number\;of\;X_{m}\left(j\right)\;where\;d\left[X_{m}\left(i\right),X_{m}\left(j\right)\right]\leq r}{N_{B}-m+1}. \end{array}$$

The normalized number of sums can be computed using

(9) $$\begin{array}{l} B^{m}\left(r\right)=\frac{1}{N_{B}-m}\sum_{i=1}^{N_{B}-m}C_{i}^{m}\left(r\right). \end{array}$$

Finally, *SampEn* was computed with

(10) $$\begin{array}{l} SampEn\left(m,r,N_{B}\right)=-\ln\left(\frac{B^{m+1}\left(r\right)}{B^{m}\left(r\right)}\right). \end{array}$$

Higuchi proposed a method to compute the fractal dimension of a signal by using sums of differences with varying inter data point intervals (delays) (Higuchi, 1988). The Higuchi dimension is frequently applied in contemporary neurophysiology and neuropathology (Kesić and Spasić, 2016) and is well known for its accuracy, speed and robustness including high linearities of the double log plots. Phase space reconstructions are not involved and therefore, the number of data points available can be restricted. Initial data points are set to *m* = 1,2,…, *k* with a delay interval *k* = 1,2,…, 30. Following data point series were constructed:

(11) $$\begin{array}{l} S_{m}\left(k\right):x\left(m\right),x\left(m+k\right),x\left(m+2k\right),\ldots \;,\;x\left(m+\left\lfloor \frac{N_{B}-m}{k}\right\rfloor k\right) \end{array}$$

The lengths *L*_*m*_(*k*) of these series, depending on the initial data points *m* and *k* were computed according to:

(12) $$L_{m}(k)=\frac{1}{k}\left\{\left(\sum_{i=1}^{\left\lfloor \frac{N_{B}-m}{k}\right\rfloor }\left|x\left(m+ik\right)-x(m+\left(i-1\right)k)\right|\right)\frac{N_{B}-1}{\left\lfloor \frac{N_{B}-m}{k}\right\rfloor k}\right\}$$

The symbol ⌊ ⌋ stands for the floor function. For each *k*, the mean length was determined by

(13) $$\begin{array}{l} L\left(k\right)=\frac{1}{k}\sum_{m=1}^{k}L_{m}\left(k\right). \end{array}$$

Finally, a double logarithmic plot of *L*(*k*) vs. *k* was constructed and the slope of a linear regression was used to compute *D*_*H*_. Values of *k* above 30 were not used because they introduced noticeable deviations of data points from the linear regression. Signals were processed as they were recorded without editing. Algorithms were implemented in the Software IQM (Kainz et al., 2015) and are available from the authors or from the IQM project page.

### Statistics

Statistics was computed with public domain software R, version 3.3.3 and RStudio software version 1.0.136 (RStudio, 2016; R Core Team, 2017). Due to small sample sizes, differences of paired samples were statistically analyzed with a two-sided median test using the R function sintv2 (Wilcox and Rousselet, 2018). This method performs very well in terms of controlling the probability of a Type I error (Wilcox, 2016). Data acquisition via video and electrode setup did not start synchronously (lag of 1–3 beats). Cross correlation (unbiased estimate, MATLAB® R2017b) was used to remove this start dependent asynchronism between the optical and electrical signal. Correlation of *CSs* with *BBIs* was computed using Spearman's rank correlation coefficient *r*_s_. Coefficients of determination *R*^2^ were computed for double log plots to estimate Higuchi dimensions.

Surrogate analysis was performed to provide further evidence of long-range nonlinear correlations in the optically measured signals and to demask possible white noise components indicated by some relatively high *SampEn* and *D*_*H*_ values. Each optically recorded signal was shuffled 50 times using IQM (Kainz et al., 2015). A total number of 5,400 surrogate data series were constructed considering nine SAN tissues, two nonlinear measures (*SampEn, D*_*H*_), two treatments (Con, ACh), three signal types (*BBI, CS, rCS*), and 50x shuffling. Following evaluation types were carried out:

SurrEval-1: Each individual experimentally gained value was tested against the normally distributed surrogate values applying a two-sided one sample Student's *t*-test.

SurrEval-2: The experimentally gained values were tested against the respective means of the shuffled signals by a two-sided median test using the R function sintv2.

SurrEval-3: The respective means of shuffled control against means of shuffled ACh signals were tested by a two-sided median test using the R function sintv2.

## Results

Linear variance measures of the beat to beat interval are dependent on absolute values and are only well suited for linear stochastic processes. Nonlinear signals with random correlations or Random walk like signals can be well investigated with scale independent measures such as the sample entropy *SampEn* or the Higuchi dimension *D*_*H*_.

### Double log plots for higuchi dimensions

**Figure 3:**
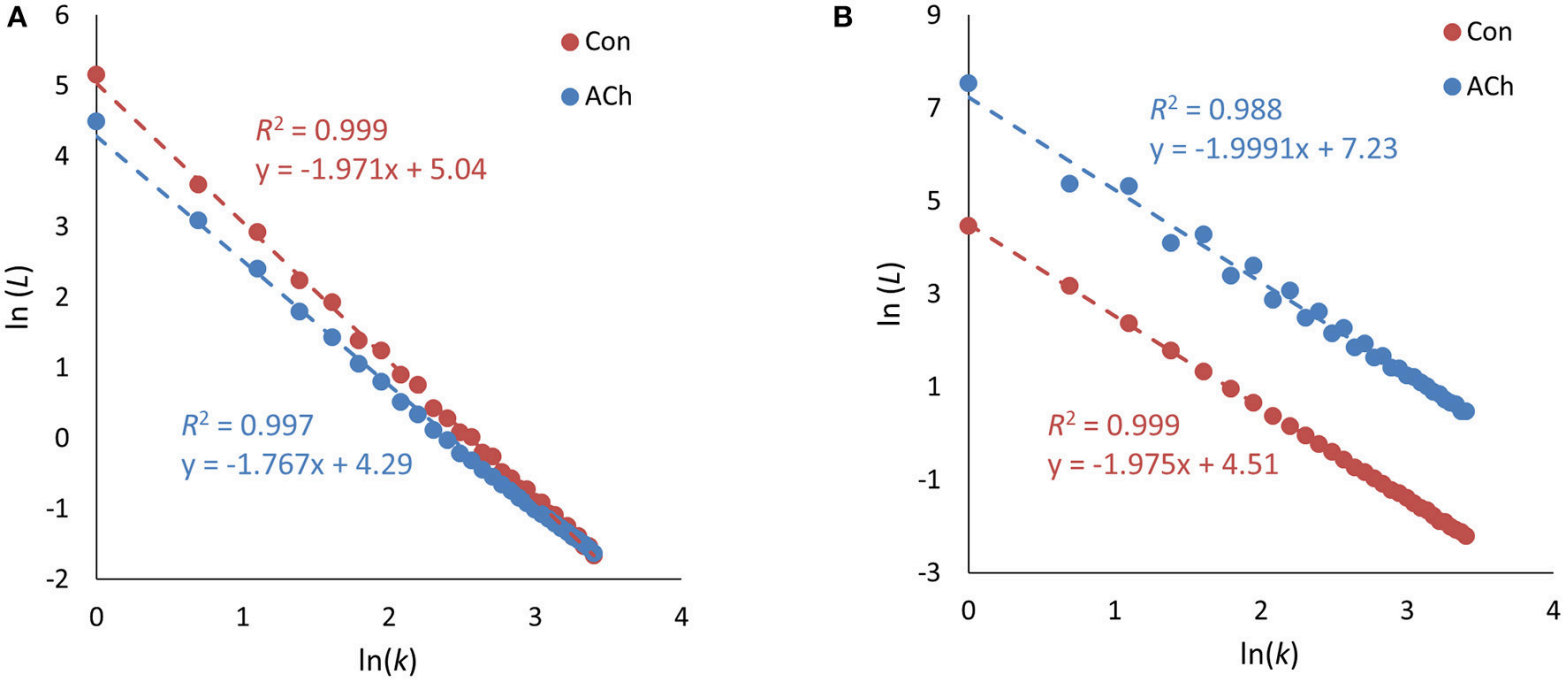
Higuchi dimension *D*_*H*_ double log plots. Data from optical recordings. **(A)** Typical double logarithmic plots of *D*_*H*_ showing very linear regressions for the control and the acetylcholine treated cases. **(B)** Another sample of double logarithmic plots showing occasional deviations from the linear regression, in this particular case for acetylcholine (blue dots).

Double log plot linear regressions for estimating the Higuchi dimensions revealed very high coefficients of determination *R*^2^ within a range of [0.959–0.999]. Sample linear regressions can be seen in Figures 3A,B for control and ACh treated cases. With this high linearity, the application of the fractal concept seems to be very appropriate and robust. Nevertheless, we found a marginally lower R^2^ for some signals (4 out of 18) which was visible as a slight wobbling of data points around the straight line (see a sample graph in Figure 3B, blue dots). This occurred for control as well as ACh treated cases. The reason was that the specific signals showed subsequent alternating values which could be interpreted as binary oscillations or negative correlations. Fractal dimensions of period doubling signals cannot be directly calculated, but it is known that periodic components in time series yield distinct and periodic differences in the double log plot (Galvez Coyt et al., 2013).

### Correlation of optically and electrically recorded beat to beat intervals

**Figure 4:**
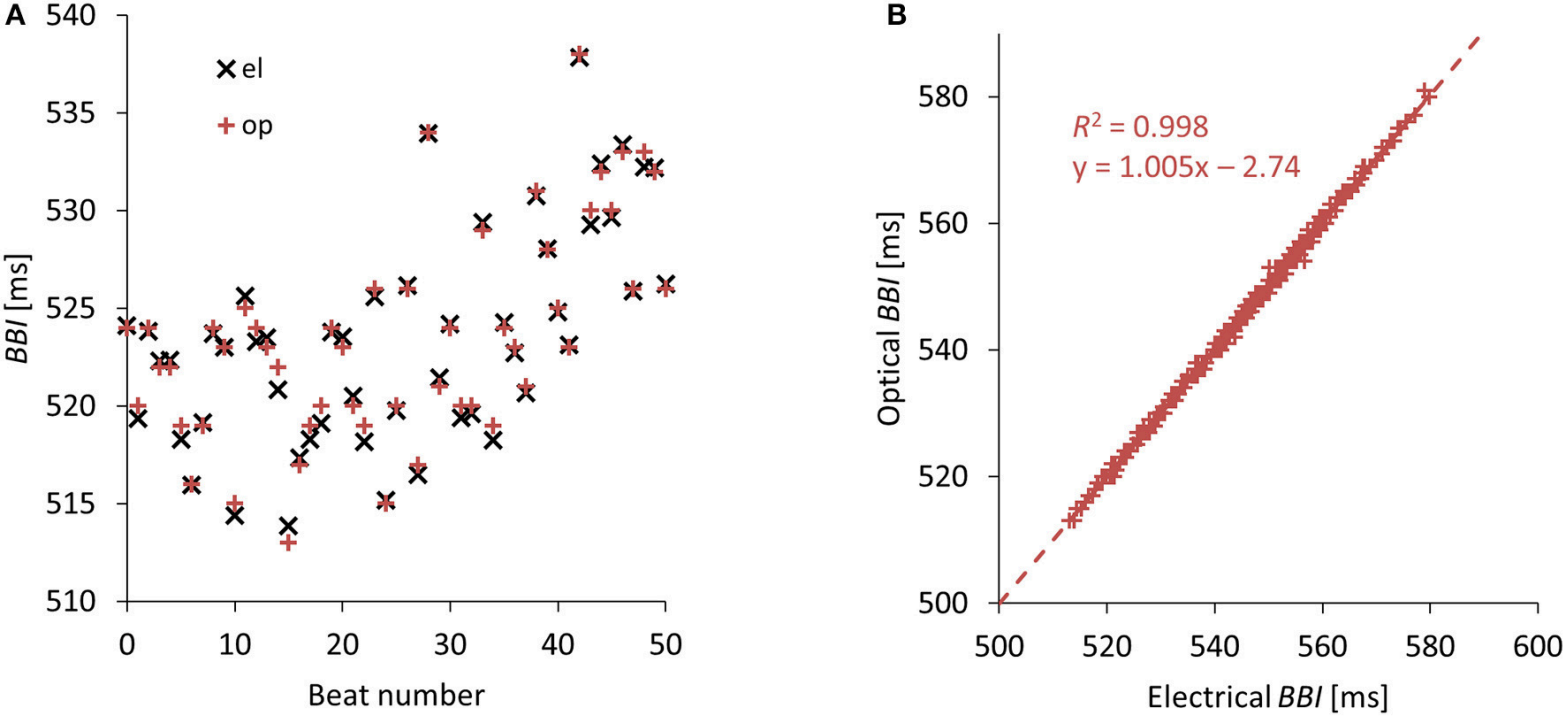
Opto-electrical correlation. **(A)** Some representative beat to beat intervals *BBIs* computed from simultaneously measured electrical and optical (video) recordings. **(B)** Regression of electrically and optically gained *BBIs* from one experiment (i.e., one mouse). The linear slope is very close to one and the coefficient of determination *R*^2^ is very high.

Optically (video) recorded values of *BBIs* correlated very well with electrically recorded values. Representative *BBI* value pairs showing just negligible differences can be seen in Figure 4A. A sample regression plot of a whole 5 min recording can be seen in Figure 4B. The slopes for all 13 experiments were in the range of [0.977–1.067] and the coefficients of determination *R*^2^ were in the range of [0.984–0.999].

**Figure 5:**
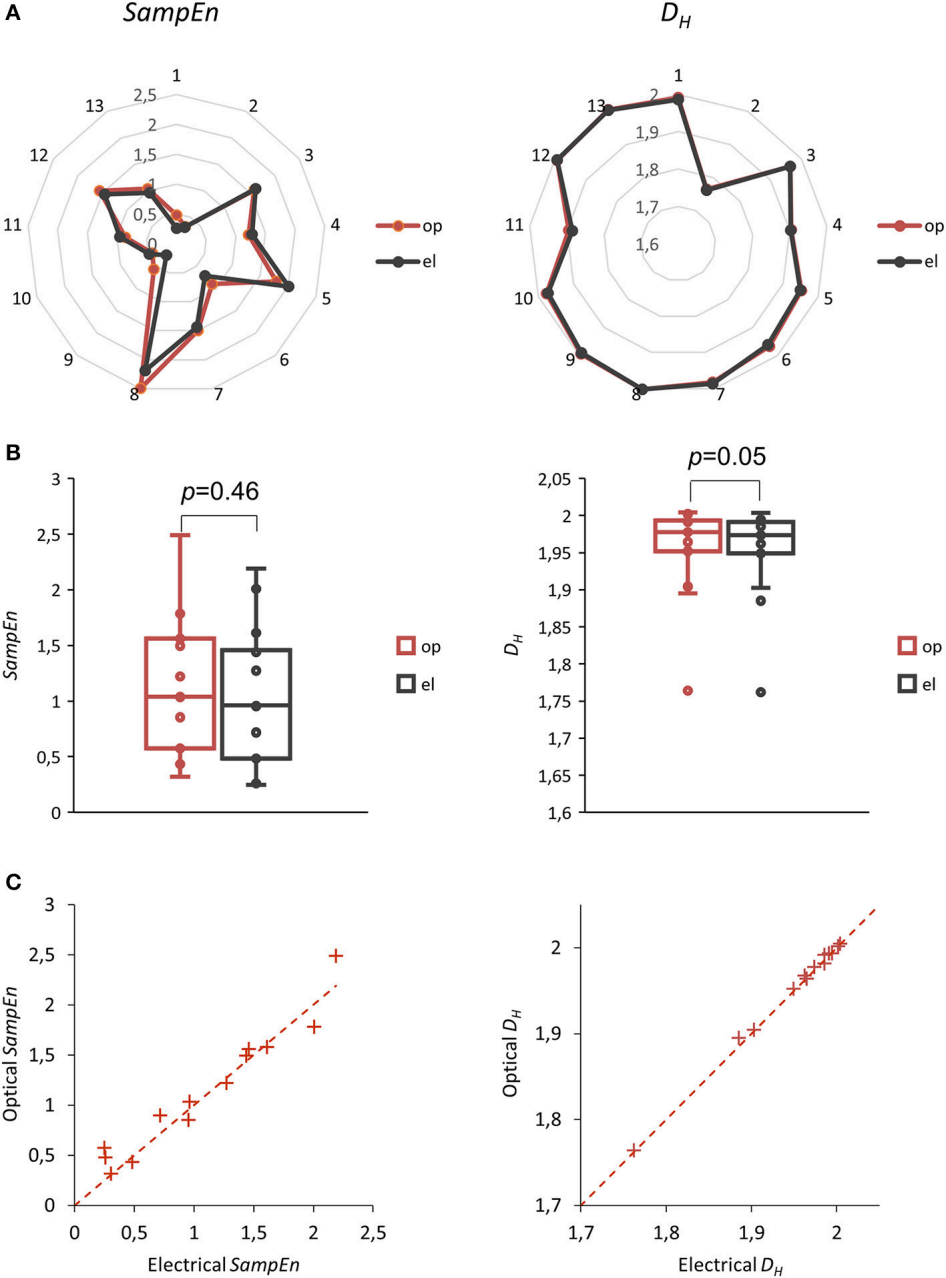
Sample entropy *SampEn* (left column) and Higuchi dimension *D*_*H*_ (right column) of beat to beat intervals *BBIs* determined optically and electrically. **(A)** Net plots showing 13 *SampEn* and *D*_*H*_ values for *BBIs*. **(B)** Box plots of these 13 experiments, *p*-values with *df* = 12, median test. **(C)** Scatterplots of these 13 experiments. Dashed lines represent theoretical correlations with the slope of one.

Additionally, we computed sample entropies and Higuchi dimensions for these 13 control samples. Results can be seen in net plots for each individual experiment in Figure 5A. Traditional box plots including the data values can be seen in Figure 5B.

*SampEn* values are very close and statistically not different (*p* = 0.46, *df* = 12). For *D*_*H*_ values a *p*-value of 0.05 (*df* = 12) indicates a possible effect. Since the corresponding median difference was very small (only in the third decimal place), we show scatterplots of data point pairs in Figure 5C. The minimal deviations from the straight line (no effect) suggest no practical relevance.

### Correlation of contraction strengths and beat to beat intervals

**Figure 6:**
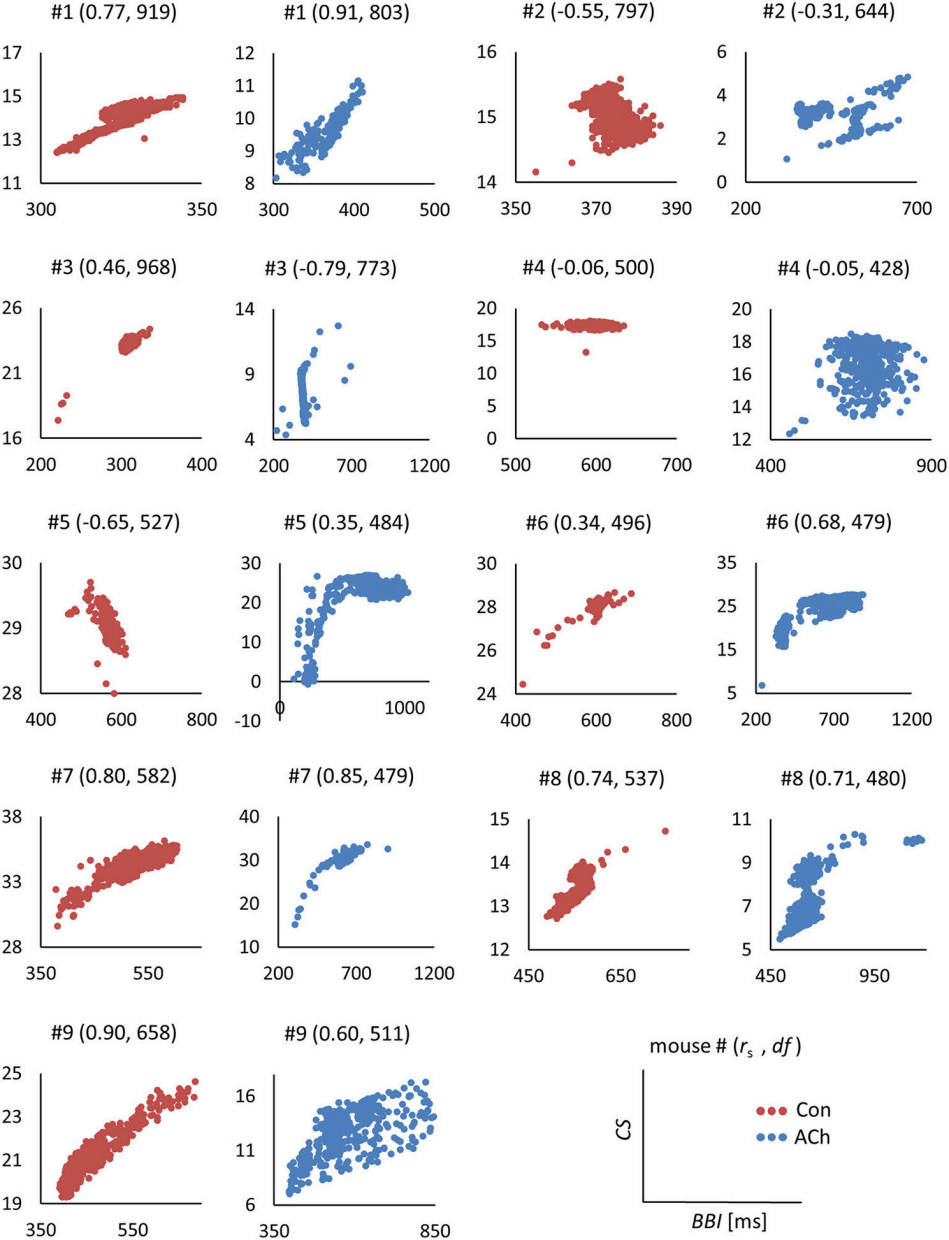
Scatterplots of contraction strengths *CSs* vs. beat to beat intervals *BBIs* in milliseconds. Data from optical recordings. Red dots correspond to control tissue, blue dots to ACh treated tissue. For each individual plot the experiment (mouse) number #, in brackets the actual Spearman's rank correlation coefficient *r*_s_ and the degrees of freedom *df* are depicted.

Scatterplots of *CSs* vs. *BBIs* in milliseconds are shown in Figure 6. Control tissue (red) and ACh treated tissue (blue) showed mostly positive correlations. A few correlations are weak and/or negative.

Additionally, scatterplots and correlations of relative contraction strengths *rCSs* vs. *BBIs* were computed. Actual plots are not shown, because the correlations were quite similar compared to Figure 6.

### Acetylcholine induced changes of beat to beat intervals and contraction strengths

**Figure 7:**
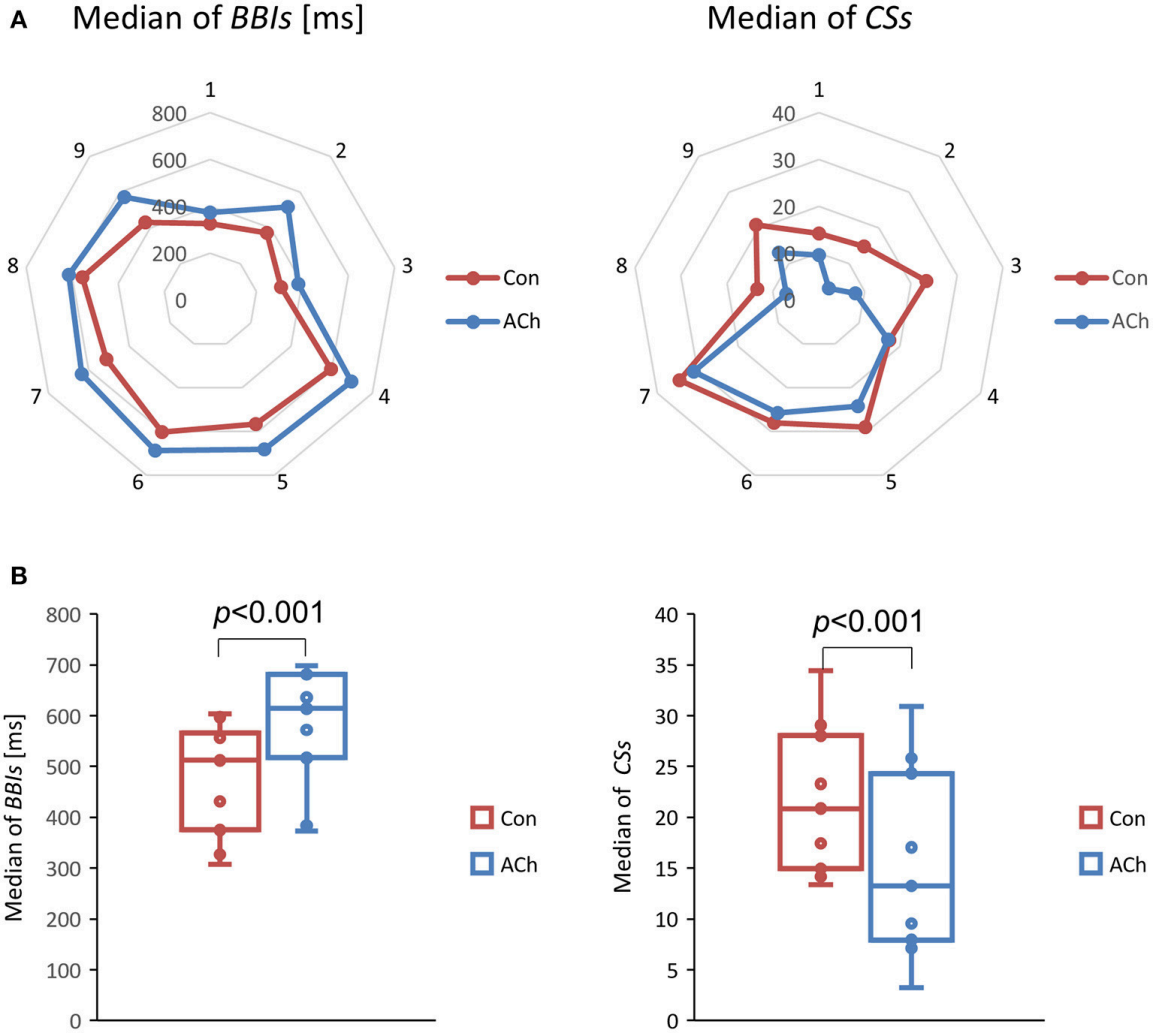
Medians of beat to beat intervals *BBIs* (left column) and contraction strengths *CSs* (right column). Data from optical recordings. The control group is depicted in red and the values for the ACh treated group in blue. **(A)** Net plots of all experiments, showing individual and pairwise values. **(B)** Box plots of all experiments show statistically significant differences between Con and ACh groups, *p* < 0,001, *df* = 8, median test.

The median beat to beat interval of the control group was 512 ms and increased to 614 ms after applying ACh. Net and box plots can be seen in Figure 7 (left column). The significant increase in the beating rate of ~20% (3 μM ACh) is in line with previously published data (Glukhov et al., 2010).

The median beat to beat contraction strength of the control group was 20.85 and decreased to 13.24 after treatment with ACh. Net- and box plots can be seen in Figure 7 (right column). The significant decrease of *CS* of ~37% is in accordance with previously published data (Kitazawa et al., 2009).

### Acetylcholine induced changes to sample entropies and higuchi dimensions

**Figure 8:**
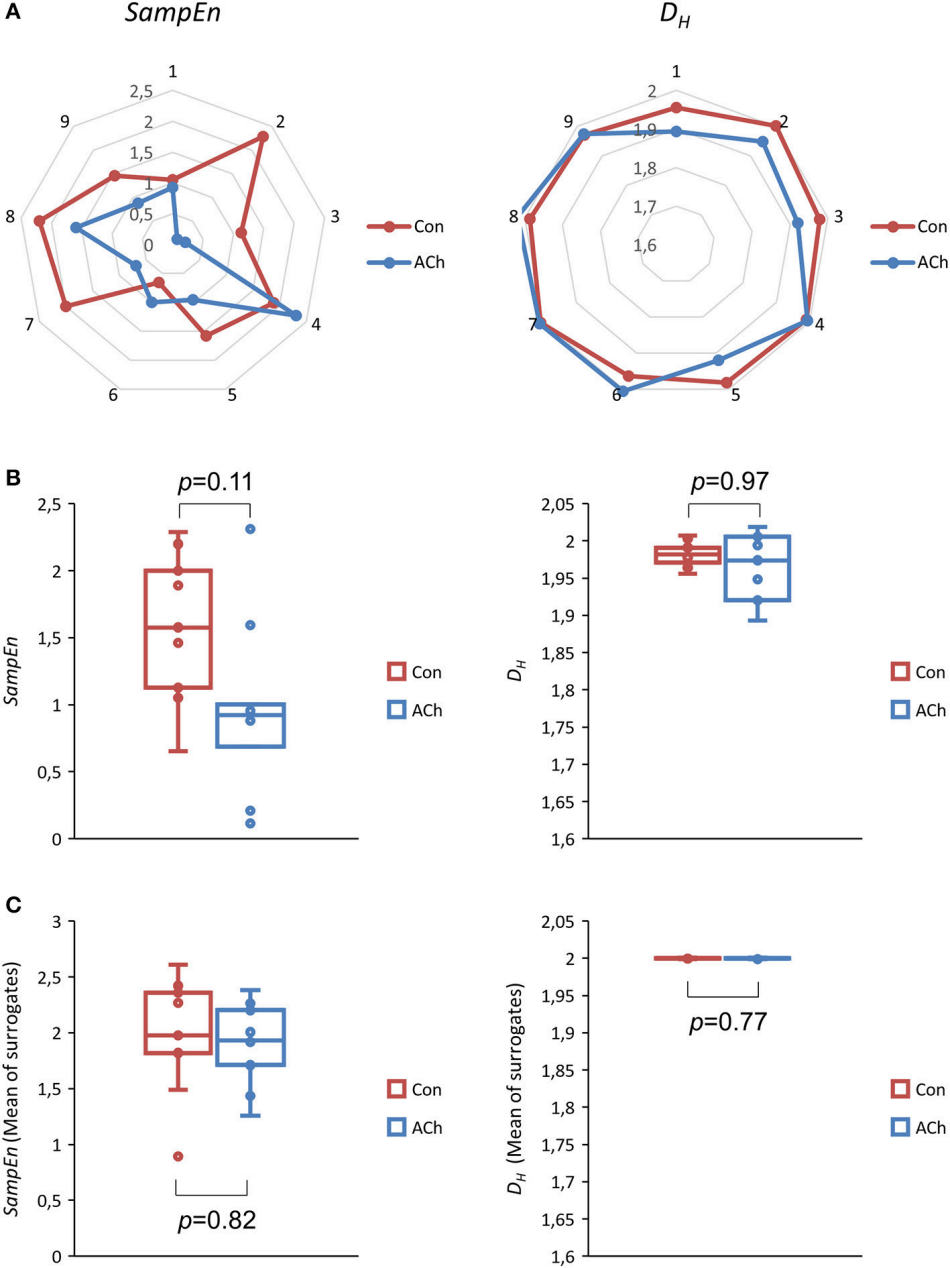
Sample entropy *SampEn* (left column) and Higuchi dimension *D*_*H*_ (right column) of beat to beat intervals *BBIs*. Data from optical recordings. The control group is depicted in red and the ACh treated group in blue. **(A)** Net plots of all experiments, showing individual and pairwise values. **(B)** Box plots of all experiments. Differences are not statistically significant (*SampEn p* = 0.11, *df* = 8, *D*_*H*_ *p* = 0.97, *df* = 8, median test). **(C)** Mean *SampEn* and *D*_*H*_ values of shuffled (50x) data series.

Figure 8 depicts net- and box plots of nonlinear measures of *BBIs*. Median values of *SampEn* decrease from 1.58 (Con) to 0.92 (ACh) and are not significantly different (*p* = 0.11, *df* = 8, Figure 8B, left column). This is also the case for *D*_*H*_ (*p* = 0.97, *df* = 8, Figure 8B, right column) which decreased from 1.98 to 1.97.

*SampEn* surrogate evaluation according to SurrEval-1 revealed that all experimentally gained values (*BBIs* for Con and ACh) were significantly lower than the shuffled ones with *p* < 0.001, *df* = 49. This agrees with SurrEval-2 showing that all experimental values were also significantly lower than the means of the corresponding shuffled ones with *p* < 0.001, *df* = 8, see Table 1. Furthermore, according to SurrEval-3 the means of *SampEn* values for shuffled signals showed no indication for statistical significance between Con and ACh, *p* = 0.82, *df* = 8, see Figure 8C.

**Table 1:** Median test of sample entropy *SampEn* and Higuchi dimension *D*_*H*_ values from optical recordings against means of 50x shuffled data series according to SurrEval-2.

**Nonlinear measure**	***BBI* Con**	***BBI* ACh**	***CS* Con**	***CS* ACh**	***rCS* Con**	***rCS* ACh**
*SampEn*	<0.001(0.36) [0.25, 0.41]	<0.001(1.05) [0.31, 1.32]	<0.001(0.72) [0.23, 1.11]	<0.001(1.50) [1.17, 1.63]	<0.001(0.47) [0.34, 1.05]	<0.001(1.73) [1.25, 2.02]
*D_*H*_*	0.05(0.02) [0.00, 0.03]	0.27(0.03) [−0.01, 0.08]	<0.001(0.03) [0.01, 0.04]	<0.001(0.09) [0.01, 0.15]	<0.001(0.02) [0.01, 0.08]	<0.001(0.11) [0.02, 0.27]

*p-values, median differences and confidence intervals (df = 8) are given for BBI, CS, rCS, and for control and ACh treatment. p-values, (median difference), [confidence interval], median test according to SurrEval-2, df=8*.

SurrEval-1 for *D*_*H*_ yielded similar results with *p* < 0.001, *df* = 49, except for some experimental values >2 in the third decimal (two cases for Con and three cases for ACh) where obviously the shuffled values could not further increase. This is well reflected by SurrEval-2 with a borderline *p* = 0.05, *df* = 8 for Con and a non-significant *p*-value for ACh (Table 1). As expected, SurrEval-3 reveals no significant difference between the groups Con and ACh, *p* = 0.77, *df* = 8, see Figure 8C.

**Figure 9:**
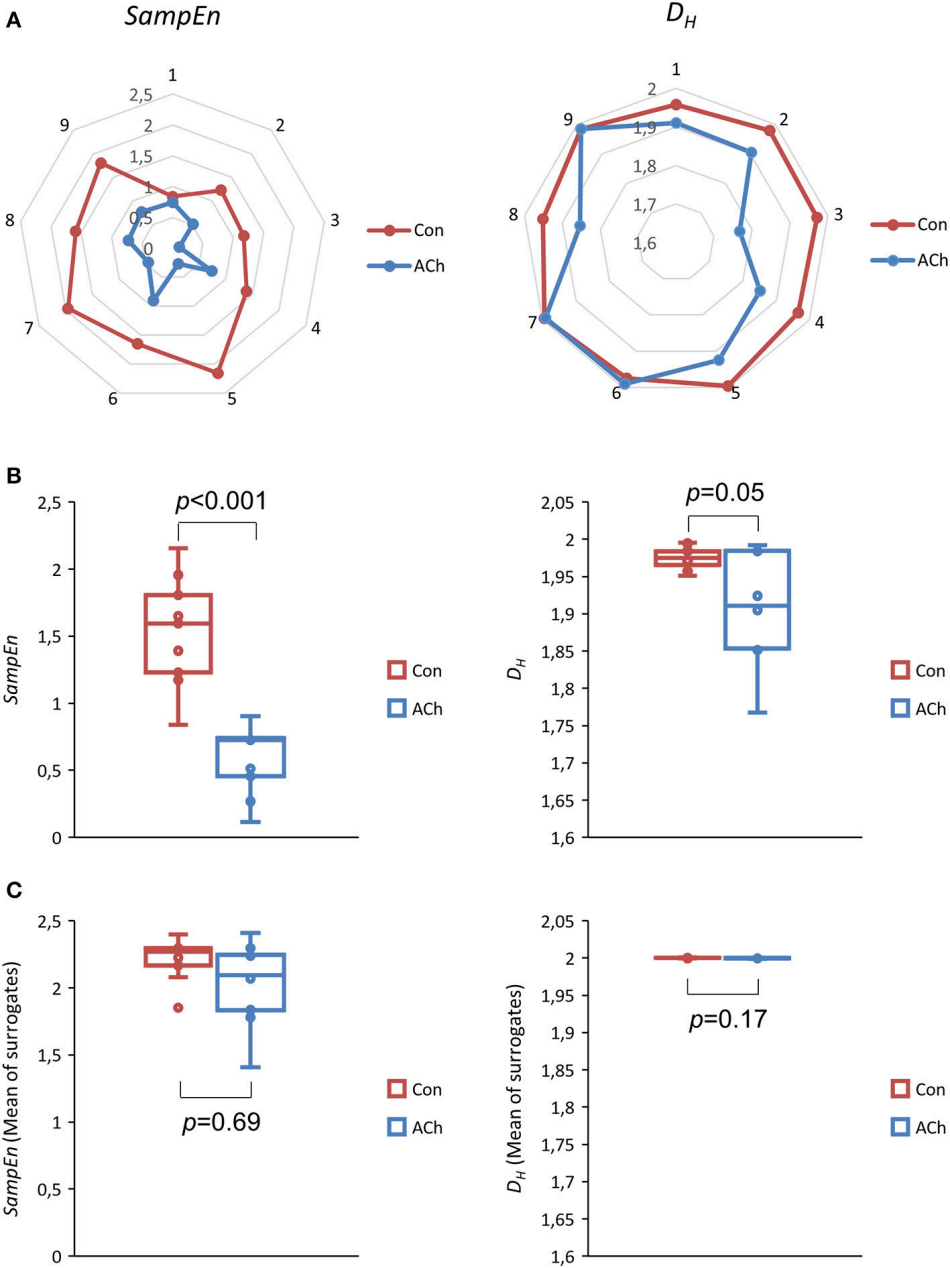
Sample entropy *SampEn* (left column) and Higuchi dimension *D*_*H*_ (right column) of contraction strengths *CSs*. Data from optical recordings. The control group is depicted in red and the ACh treated group in blue. **(A)** Net plots of all experiments, showing individual and pairwise values. **(B)** Box plots of all experiments. The difference in *SampEn* is statistically significant, *p* < 0.001, *df* = 8, median test. The difference in *D*_*H*_ is statistically borderline (*p* = 0.05, *df* = 8). **(C)** Mean *SampEn* and *D*_*H*_ values of shuffled (50x) data series.

Figure 9 shows net and box plots of nonlinear measures of the proposed *CSs*. Median values for *SampEn* show a significant difference between Con and ACh (*p* < 0.001, *df* = 8), namely a decrease from 1.60 (Con) to 0.72 (ACh) which can been seen in Figures 9A,B, left column. Median values for *D*_*H*_ tend to decrease slightly (Figure 9B, right column) as indicated by a borderline *p*-value of *p* = 0.05 (*df* = 8). In detail median values decrease from 1.97 (Con) to 1.91 (ACh).

For *SampEn*, all three *CS* surrogate evaluations yielded consistent results regarding nonlinear patterns in the measured signals, since shuffled values were always statistically higher (SurrEval-1: *p* < 0.001, *df* = 49, and SurrEval-2: Table 1) and the difference between Con and ACh vanished compared to the experimental case (SurrEval-3: Figure 9C). Now, SurrEval-1 for *D*_*H*_ yielded no exception with *p* < 0.001, *df* = 49 and all three surrogate evaluations are again consistent (SurrEval-2: Table 1 and SurrEval-3: Figure 9C).

**Figure 10:**
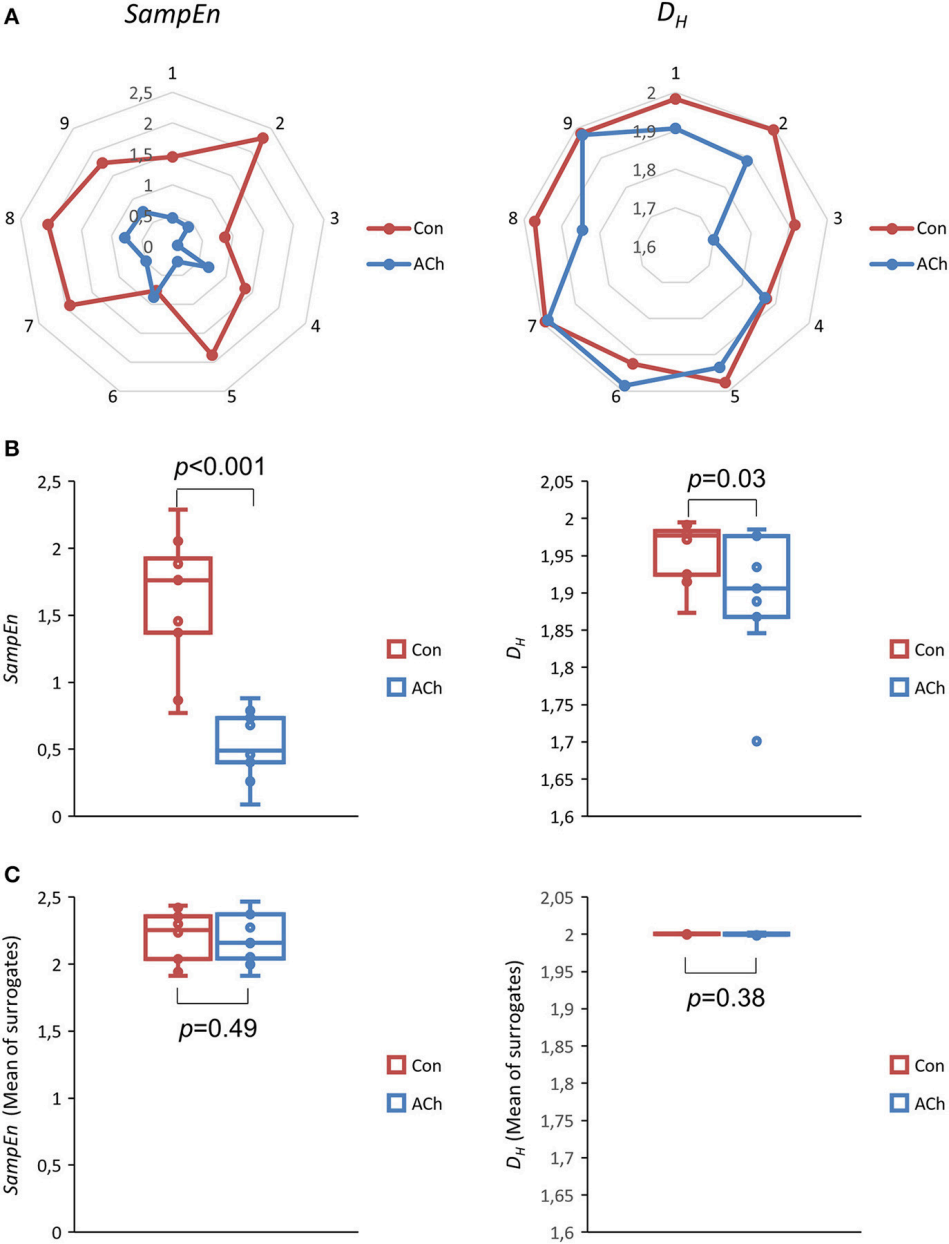
Sample entropy *SampEn* (left column) and Higuchi dimension *D*_*H*_ (right column) of relative contraction strengths *rCSs*. Data from optical recordings. The control group is depicted in red and the ACh treated group in blue. **(A)** Net plots of all experiments, showing individual and pairwise values. **(B)** Box plots of all experiments. The differences in *SampEn* and *D*_*H*_ are statistically significant, *p* < 0.001, *df* = 8 and *p* = 0.03, *df* = 8 respectively, median test. **(C)** Mean *SampEn* and *D*_*H*_ values of shuffled (50x) data series.

Finally, Figure 10 depicts net and box plots of nonlinear measures of the proposed *rCSs*. For *SampEn*, the decrease and the significance of *rCS* is similar to *CS* with median values from 1.76 (Con) to 0.49 (ACh) and with *p* < 0.001, *df* = 8 (Figures 10A,B, left column). The decrease of *D*_*H*_ values is slightly more pronounced for *rCS* compared to *CS* with median values from 1.98 (Con) to 1.91 (ACh) and now exceeds the 95% statistical significance level with *p* = 0.03, *df* = 8 (Figure 10B, right column).

Surrogate analyses for *rCs* are again consistent for both, *SampEn* and *D*_*H*_, indicating nonlinear long-range correlations. Shuffled values are always statistically higher (SurrEval-1: *p* < 0.001, *df* = 49, and SurrEval-2: Table 1) and the difference between Con and ACh vanishes compared to the experimental case (SurrEval-3: Figure 10C).

## Discussion

Beat to beat intervals are commonly investigated in order to detect nonlinear correlations in time signals. This study proposes an optical method, particularly, a high-speed video technique to detect mechanical contractions of the heart tissue. Usual video recordings with frame rates of about 30 fps or software triggered acquisitions are a convenient way for spectral analyses or computing beating frequencies (Kojima et al., 2006; Chan et al., 2009; Fassina et al., 2011; Hsiao et al., 2013; Ahola et al., 2014). But obviously, video frame rates must be higher for high beating rates (De Luca et al., 2014) or accurate detections of beating events (Stummann et al., 2008). Our high-speed video recordings allowed us to extract beat to beat intervals *BBIs* as well as the contraction strengths *CSs* and the relative contraction strengths *rCSs*, because the average gray value of an image was directly proportional to the mechanical contraction. Variation analyses with two distinguished nonlinear measures, the sample entropy *SampEn* and the Higuchi dimension *D*_*H*_, revealed that this video technique is able to produce consistent results for *BBIs* as well as for *CSs* and *rCSs*.

The detection of the baseline (relaxed tissue) may be prone for errors such as measurement noise or optical drifts during the recording and thus, we proposed the second contraction parameter *rCS*. This is basically the varying contraction content of the signal, without the absolute value, drift or offset. *SampEn, D*_*H*_ and other scale independent nonlinear measures or fractal dimensions are not dependent on absolute values and consequently, *rCS* is an appropriate and very promising parameter for variance analyses.

To our knowledge, studies of BBV using isolated SAN tissue are very limited. Since no consensus exists to classify possible physiological artifacts (e.g., ectopic beats) in this *in vitro* preparation, we analyzed the original signals without any editing that could lead to a loss of valuable information.

We observed that ACh changed the beating behavior of the sinus node tissue by significantly reducing beating frequency as well as contraction strength, which is in accordance to previously published data (Kitazawa et al., 2009). Application of *SampEn* and *D*_*H*_, two frequently used nonlinear measures for time signal variations, revealed a significant change of variabilities in the contraction strength but not in the beat to beat interval. The observed reduction of nonlinear measures indicates that the contraction process estimated by *CS* and *rCS* becomes more regular in the SAN tissue after ACh application. The spontaneous activity of pacemaker cells in the SAN tissue is based on two tightly linked clocks referred to as calcium and membrane clock (Lakatta and DiFrancesco, 2009). Both clocks exhibit inherent random components which arise from stochastic opening and closing of transmembrane ion channels (Krogh-Madsen et al., 2017) in the case of the membrane clock and from spontaneous stochastic calcium release via sarcoplasmic ryanodine receptors (Yaniv et al., 2014b) in the case of the calcium clock. The spontaneous calcium release in turn activates the sodium-calcium exchanger thereby triggering the action potential upstroke and subsequently a massive calcium release from sarcoplasmic reticulum, thus coupling excitation with contraction. ACh is an important modulator of SAN beating frequency as well as of contraction strength, particularly in the adjacent atrial tissue (Okada et al., 2013). Activation of muscarinic receptors by ACh causes multiple effects on the membrane and the calcium clock via G-protein coupled signaling ultimately reducing beating frequency and contractility (Harvey and Belevych, 2003). This is in line with our results. The physiological mechanisms underlying the observed increase in contraction strength regularity by ACh in our study are currently unknown. Theoretically, a reduced randomness in membrane and/or calcium clock as well as in the contraction process itself could account for our observation. It is noteworthy that in our study ACh increases *CS* regularity but not *BBI* regularity. This may be due to the fact that the beating behavior in the time domain is determined solely by sinus node pacemaking, whereas *CS* regularity may also depend on the effect of ACh on atrial tissue present in our preparations.

Studies on ACh effects on SAN cells/tissue using nonlinear measures are very scarce. Yaniv et al (Yaniv et al., 2014a) investigated the beating rate variability at different levels of integration from the heart *in vivo* to single pacemaker cells by linear (coefficient of variation) and nonlinear (approximate entropy, power law and detrended fluctuation analysis) measures. Their results show that beating interval regularity increased in the order *in vivo*, denervated heart, isolated SAN tissue, but decreased again in single pacemaker cells. However, single SAN cells showed fractal-like behavior only to a small percentage. Carbachol, a parasympathomimetic drug, decreased regularity of beating intervals of single SAN cells. Since this group analyzed the effect of parasympathetic stimulation on beating behavior only in the time domain and at the single cell level, a direct comparison to our results does not seem to be reasonable. Clearly, further studies are needed to elucidate the underlying physiological mechanisms of muscarinic stimulation on nonlinear measures in SAN cells/tissue.

Compared to commonly used mechanical force transducer measurements (Kihara and Morgan, 1991; Torres and Janssen, 2011; Koyani et al., 2017), the high-speed video technique used in this study seems to be an appropriate, contact-free tool to quantify changes in contraction strength variability. The SAN preparation represents a very sensitive and fragile tissue which could be easily damaged by hooks or threads of mechanical transducers. Moreover, SAN tissue may not be very suitable for mechanical force measurements because forces developed by the low tissue mass are very small leading to low signal amplitudes and hence low signal to noise ratios. The suggested video method does not provide absolute values of contraction forces. Absolute values are certainly a prerequisite for linear analyses but not for nonlinear investigations of variabilities that are *per se* independent of the absolute value. The electrical and the contractile processes underlying the measured *BBIs* and *CSs* are not reducible to each other, although tightly linked. Thus, the measured video signal provides information about these two distinct processes and consequently allows a more comprehensive characterization compared to frequently used electrode techniques.

For optically computed *SampEn* values, all surrogate evaluations strengthen the experimental findings that long-range correlations are present in *BBI, CS*, and *rCS* signals. Shuffled values are always statistically higher compared to experimentally obtained values and the difference between control and ACh treated tissue vanishes compared to the experimental case. Hence, the investigated physiological signals contain inherent nonlinear patterns in the interval and the contraction strength domains, justifying the application of the chosen nonlinear measures. Furthermore, the increase of regularity due to ACh (lower *SampEn* and *D*_*H*_ values) seems to be caused by deterministic and not by random processes.

The surrogate analyses for *D*_*H*_ values agree very well to *SampEn* evaluations, except for *BBI* signals (see Table 1). Particularly, some experimental values were already close to two, implicating a high degree of underlying random processes. Obviously, data time series shuffling did not reveal any significant changes. Distinct *D*_*H*_ and *SampEn* surrogate results concerning *BBIs* imply different sensitivities to underlying random and deterministic physiological mechanisms. This may indicate that the Higuchi dimension and not the sample entropy is able to discriminate differences between interval and contraction strength signals, but further investigations are needed to corroborate this assumption. In order to rule out that high *D*_*H*_ values close to two were method specific, we additionally performed a detrended fluctuation analysis DFA (Peng et al., 1994; Goldberger et al., 2002). DFA characterizes white noise with α = 0.5 and Brownian noise with α = 1.5.The maximal window length was set to 30, comparable to *k* = 30 for *D*_*H*_. The medians of all control cases including *BBI, CS*, and *rCS* (*n* = 27) were 1.98 for *D*_*H*_ and 0.61 for α (DFA) confirming the high degrees of randomness in the signals. Thus, the decreased *D*_*H*_ values under ACh treatment indicate a change from very low correlations to increased long-range correlations and self-affine processes.

Complexity can be defined as the presence of long-range correlations, arising from nonlinear interaction dominated dynamical processes being neither totally regular nor totally irregular (Van Orden et al., 2011). This concept has been successfully applied to discriminate healthy and pathological conditions, where a breakdown of long-term correlations and an according change in fractal dimension has been observed (Goldberger et al., 2002). In our case, SAN tissue preparations show a high degree of irregularity near white noise indicating a low complexity without long-range correlations or self-organizing mechanisms. This may be due to a loss of multiple and interwoven communication pathways and nonlinear dependencies present in the intact heart but not in the tissue preparation. This is supported by ACh as a relevant external stimulus that changes the interaction dominated dynamical system by reducing the degrees of freedom and by introducing long-range correlations, multiplicative interactions and feedback. ACh may be interpreted as a control parameter for the system. Phase transitions or bifurcations, dependent on control parameters exceeding critical values, may also play an important role for our tissue preparation, but at this stage further investigations are necessary to justify such interpretations.

In conclusion, the described technique represents a reliable, easy handling and long-lasting recording method, from which beating rate variabilities and contraction strength variabilities can be assessed.

## Author contributions

HA and SS are joint first authors. HA, SS, KZ-P, and BP designed the study. SS and PL dissected SAN tissues from mouse hearts. SS and ÁD carried out video experiments. SS and RA carried out electrical recordings. HA, MM-R, and ÁD developed mathematical algorithms. HA, SS, ÁD, and PL performed video analyses. RA analyzed electrical recordings. KZ-P and HA computed nonlinear parameters and statistical tests. HA, SS, and KZ-P wrote the manuscript. SS, RA, BP, KZ-P, HA, and MM-R revised the manuscript. HA and SS prepared the figures. All authors read and approved the manuscript.

## Data availability statement

*BBI, CS*, and *rCS* data series for each experiment (Con and ACh treated) are provided as csv file in the supplement.

### Conflict of interest statement

MM-R was employed by KML vision OG, Graz, Austria. KML vision OG did not sponsor this study. The other authors declare that the research was conducted in the absence of any commercial or financial relationships that could be construed as a potential conflict of interest. The handling Editor declared a past co-authorship with one of the authors HA.
